# Mortality of older persons living alone: Singapore Longitudinal Ageing Studies

**DOI:** 10.1186/s12877-015-0128-7

**Published:** 2015-10-15

**Authors:** Tze Pin Ng, Aizhen Jin, Liang Feng, Ma Shwe Zin Nyunt, Khuan Yew Chow, Lei Feng, Ngan Phoon Fong

**Affiliations:** Department of Psychological Medicine, Gerontology Research Programme, National University Health System, Yong Loo Lin School of Medicine, National University of Singapore, Singapore, Singapore; National Registry of Diseases Office (NRDO), Health Promotion Board, Singapore, Singapore; Department of Psychological Medicine, Gerontology Research Programme, National University of Singapore, NUHS Tower Block, 9th Floor, 1E Kent Ridge Road, Singapore, 119228 Singapore

**Keywords:** Ageing, Living alone, Health status, Mortality

## Abstract

**Background:**

We investigated the association of living alone with mortality among older persons, independently of marital, health and other factors, and explored its effect modification by age group, sex, marital status and physical functional disability.

**Method:**

Using data from 8 years of mortality follow up (1 September 2003 to 31 December 2011) of 2553 participants in the Singapore Longitudinal Ageing Studies (SLAS) cohort, we estimated hazard ratio (HR) of mortality associated with living alone using Cox proportional hazard models.

**Results:**

At baseline, 7.4 % (*N* = 189) of the participants were living alone, and 227 (8.9 %) died during the follow up period. Living alone was significantly associated with mortality 1.66 (95 % CI, 1.05–2.63), controlling for health status (hypertension, diabetes, chronic lung disease, stroke, heart disease, kidney failure, IADL–ADL disability and depressive symptoms), marital status and other variables (age, sex, housing type). Possible substantive effect modification by sex (p for interaction = 0.106) and marital status (p for interaction <0.115) were observed: higher among men (HR = 2.36, 95 % CI, 1.24–4.49) than women (HR = 1.14, 95 % CI, 0.58–2.22), and among single, divorce or widowed (HR = 2.26, 95 % CI, 1.24–4.10) than married individuals (HR = 0.83, 95 % CI, 0.30–2.31).

**Conclusion:**

Living alone was associated with increased mortality, independently of marital, health and other variables. The impact of living alone on mortality appeared to be stronger among men and those who were single, divorced or married.

## Background

Increasing numbers of older persons worldwide are living alone. As much as 50 % of older women in countries in Europe and North America live alone [1], and although the figures are considerably lower in Asia at less than 10 %, an increasing trend is unmistakable [2]. Living alone as a proxy measure of social isolation and the lack of social support is of practical interest and importance because of its potential negative impact on health. A substantial body of evidence supports a link between social isolation and emotional stress, adverse health behaviour, poor access to health care, and adverse health outcomes [3–8].

However, studies of the association of social isolation with increased mortality have yielded mixed findings [9–31]. Although some studies have found that living alone or loneliness was associated with increased mortality [9–19], other studies have found that living alone did not have a detrimental impact on survival [20–23], or paradoxically, was associated with decreased risk of mortality [24–26]. In some population studies with findings of null or negative associations, older persons who live alone, compared to their counterparts who live with others, were found to be in no worse physical health and functional status [20, 23].

Older persons living alone tend to be over–represented by those who are un–married, widowed or divorced, among whom negative health behaviours and status are more frequent [32–34]. Notably, a meta–analysis of 53 independent studies shows that being widowed, divorced, and never married was significantly associated with greater risk of death [35]. Not all studies of the impact of living alone on mortality have controlled for the effect of marital status, (17, 18) and therefore the independent effect of living alone apart from marital status appears unclear.

Mixed findings of the impact of living alone on mortality may reflect heterogeneity of effect across different studies of populations that varied by age, sex, economic and marital and health status. The significant heterogeneity of effect due to age is amply shown in a meta–analysis which found social isolation being more predictive of death in samples with an average age younger than 65 years [27]. This is corroborated by a small number of studies which stratified their analyses by age groups, and found living alone to be associated with higher mortality among younger participants, but among older persons (aged 75 or 80 years and over), living alone was not associated with increased mortality [17, 18, 20]. Other studies have found that living alone was associated with increased mortality more strongly among men than women, or only among men but not women [20, 28–30]. Some authors argued that living alone was not associated with increased mortality because older persons who live alone are more likely to be a self–selected population of those who are in good health and independence in the basic activities of daily living, (28, 31) which are known to strongly predict survival. Older persons with physical functional disability and therefore high mortality are less likely to be living alone [24]. The association of living alone with mortality may thus be moderated by age, sex, socioeconomic, marital and health status.

Prior studies of mortality associated with older persons living alone have mostly been conducted in economically advanced countries in the West and Japan. Few studies have been reported in Asian countries (excluding Japan) which have the fasting ageing populations in the world. In Singapore (population 5 million), the proportion of older persons aged 65 and above is expected to increase from 12 % in 2014 to 20 % in 2030. At the same time, the number of older persons living alone (most of whom are single, divorced or widowed) is expected to more than double in 2030. (Population Trends 2013 report of the Department of Statistics) This population–based study investigated the association of living alone with overall mortality among older persons in Singapore using data from 8 years follow up of the Singapore Longitudinal Ageing Studies (SLAS) cohort, from September 2003 to 31 December 2011. We assessed the baseline self–selection characteristics of age, sex, socioeconomic, marital and health and functional status associated with living alone in this Asian population cohort, and determined the independent effect of living alone on mortality controlling for the confounding effects of socio–demographic, marital and health status. We assessed possible heterogeneity of effect of living alone on mortality by exploring statistical interactions and describing the association of living alone with mortality among subgroups of older and younger participants, men and women, those with and without physical functional disability and married and unmarried (single, widowed, divorced).

## Methods

### Study population

Between September 2003 and December 2005, a whole population of older adults aged 55 years and above who were Singaporean residents in contiguous precincts in the South East region of Singapore were identified from a door–to–door census and invited to participate in the Singapore Longitudinal Ageing Study (SLAS). The response rate was 78.2 %. All participants provided written informed consent. The study was approved by the Institutional Review Board of National University of Singapore. Full details of the survey procedures and baseline study variables and data collection are described in previous publications [36]. In this study, baseline information on demographic variables, medical history, physical functional status and mental health status were collected from face–to–face interviews conducted by trained nurses using structured questionnaires at the participants’ home.

### Baseline measurements

#### Living arrangement

Respondents were asked during the interview “Who do you live with?” The close–ended responses include (1) live with spouse, son, daughter, grand–children, other relatives or friends or others, (‘living with others’) or (2) ‘live alone’.

### Covariates

#### Housing type

A housing index of socioeconomic status was constructed based on the participant’s report of housing types: low end, 1–3 room public housing apartments or higher end public housing with 4 or more rooms, or private housing (apartments, condos, landed housing). This has been shown to be a robust indicator of socioeconomic status for the Singaporean population [37].

#### Medical comorbidities

The number of medical comorbidities was obtained from self–reported history of medical conditions diagnosed by a doctor and corroborated by the report of specific drugs, diagnostic or interventional procedures, and laboratory test results. The presence of hypertension was defined by self–report of hypertension with anti–hypertensive medication use, or blood pressure measurement (untreated hypertension: systolic blood pressure (SBP) ≥ 140 mmHg or diastolic blood pressure (DBP) ≥ 90 mmHg); diabetes mellitus was defined by self–report of diabetes on anti–diabetic medications, or by elevated fasting blood sugar (untreated diabetes: fasting blood glucose ≥ 7.0 mmol/L); stroke from self–report of stroke or transient ischemic attack; myocardial infarction, atrial fibrillation and congestive heart failure from self–report, ECG evidence and/or use of appropriate medications, or interventional procedures such as PTCA or CABG; lipid abnormalities from self–report of high cholesterol and use of lipid lowering drug use or abnormal lipid panel results (Total cholesterol ≥ 6.5 mmol/L or low density lipoprotein (LDL) ≥ 4.1 mmol/L or triglyceride (TG) ≥ 2.3 mmol/L or high density lipoprotein (HDL) < 1.0 mmol/L) [38]. Other co–morbid conditions included self–reports of cataracts, asthma, chronic obstructive lung disease (COPD), arthritis, hip fracture and other problems. Multiple comorbidity was defined as 2 or more comorbid medical conditions (versus one or no medical conditions).

#### Depression

The presence of depressive symptoms was determined by the 15–items Geriatric Depression Scale (GDS–15) with scores ranging from 0 to 15 [39]. The GDS–15 is a valid and reliable screening tool for depression. In validation studies of Singaporean older adults [40], its Cronbach’s alpha was 0.80, intraclass coefficients of test–retest reliability was 0.83 and inter–rater reliability was 0.94. Using a GDS cutoff of > = 5 denoting clinically significant depressive symptoms, the GDS–15 has a sensitivity of 0.97 and specificity of 0.95 (area under curve of 0.98) for determining major depressive disorder according to DSM–IV criteria.

#### Functional status

Self–reported physical functional status was assessed using 10 items from the Barthel Index of Activities of Daily Living (ADL) [41] (needing assistance in feeding, bathing, toileting, grooming, etc.) and 8 items in the Lawton Instrumental Activities of Daily Living (IADL) Scale [42], (needing assistance in using telephone, taking medicine, travelling, managing money, etc.) which has been previously validated for use in the local population [43, 44]. Likert scores of IADL and BADL (0, 1, 2) were summed with maximum score denoting no disability, and summed scores less than the maximum score, denoting at least one disability.

### Mortality follow up

The mortality status of the SLAS participants during follow up from baseline up to 31 December 2011 was determined by using the participants’ unique National Registration Identity Card (NRIC) number for computerized record linkage with the National Death Registry through the National Disease Registry Office (NDRO) of the Ministry of Health.

### Statistical analysis

Comparison of baseline characteristics between study participants living alone and participants living with others were performed with significance testing using *T*–test for continuous variables and *χ*^2^ for categorical variables. Survival analyses with Kaplan–Meier plots of survival were performed on time to event (death) data, which were censored at data of death or on 31 December 2011. Univariate and multivariate Cox proportional hazard regression analyses with testing of proportional hazard assumption were used to estimate hazard ratio (HR) with 95 % confidence intervals (95 % C.I.) of mortality rate associated with age, sex, housing type, medical comorbidities (history of hypertension, diabetes, heart disease, chronic lung disease, stroke, kidney failure), functional disability, depressive symptoms, marital status, and living alone. The hazard ratio for living alone (versus living with others) was estimated in a series of hierarchical regression models adjusting sequentially for demographic and economic factors (age, sex, housing type), marital status, and health factors (medical morbidities, functional disability, and depressive symptoms) in the whole population sample. To assess possible non–homogeneity of effect of living alone on mortality, we explored statistical interactions of living alone with age group, sex, housing type, marital status, and IADL–BADL disability. We presented stratified data to describe mortality associated with living alone for sub–populations defined by age, sex, house type, marital status, and physical functional status. To obviate poor power or sensitivity in significance testing to detect interactions that are substantively important, interactions with a *p*–value lower than 0.15 were deemed to indicate substantive heterogeneity of effects that should be further investigated.

## Results

From among a total of 2804 participants, we excluded a small number of 193 non–Chinese participants and analyzed the data of 2553 Chinese participants with available data on living arrangement in the present study. Participants lacking data on living arrangement (*N* = 58) were very similar in almost all characteristics to those who were included in the analysis.

Among 2553 SLAS participants at baseline, 7.4 % (*N* = 189) were living alone. Participants who lived alone compared to their counterparts were more likely to be older, female, living in low–end public housing, and single, widowed or divorced. (Table 1) Participants who lived alone did not differ significantly from those who lived with others on the mean number of chronic medical conditions or IADL–ADL disability, but they had significantly more depressive symptoms.

**Table 1:** Baseline characteristics of SLAS cohort in 2003–2004 by living arrangement status

	Living with others	Living alone	*P*
Total	2364 (92.6)	189 (7.4)	
Age (mean, SD)	67.6 (7.4)	65.9 (7.7)	0.002
<75	2063 (87.3)	158 (83.6)	0.33
75–84	255 (10.8)	27 (14.3)	
85+	46 (2.0)	4 (2.1)	
Sex: Female	1470 (62.2)	148 (78.3)	<0.0001
Male	894 (37.8)	41 (21.7)	
Housing type:			
Low end (1–3 room) public housing	652 (27.6)	121 (64.0)	<0.0001
Higher end public and private housing	1712 (72.4)	68 (36.0)	
Marital status: Single, divorce, widowed	487 (20.6)	157 (83.1)	<0.0001
Married	1877 (79.4)	32 (16.9)	
No. medical comorbidities, mean (SD)	2.31 (1.5)	2.42 (1.4)	0.289
≤1	761 (32.2)	54 (28.6)	
≥2	1603 (67.8)	135 (71.4)	0.304
History of hypertension (yes)	1313 (55.1)	112 (59.3)	0.270
History of Diabetes (yes)	405 (17.1)	34 (18.0)	0.764
History of Chronic Lung Disease (yes)	89 (3.8)	3 (1.6)	0.124
History of Stroke (yes)	91 (3.9)	3 (1.6)	0.112
History of Heart Disease (yes)	209 (8.9)	18 (9.5)	0.758
History of Kidney Failure (yes)	15 (0.6)	2 (1.1)	0.491
IADL-ADL % (at least 1 item) disability	585 (24.8)	42 (22.2)	0.438
Depressive symptoms, DS (GDS > =5)	295 (12.5)	41 (21.7)	0.0003

Figures shown are n (%) or mean (SD)

Up to 31 December 2011, a total of 227 (8.9 %) participants died. Table 2 shows the expected increased mortality associated with older age, male sex, residence in low–end public housing, being single, divorced or widowed, medical morbidities, IADL–BADL disability, and depressive symptoms in univariate analyses. Compared to subjects living with others, participants who lived alone showed significantly higher mortality rates. (Table 3) The hazard ratio adjusted for age, sex, housing type, marital status, history of hypertension, diabetes, heart disease, chronic lung disease, stroke, kidney failure, IADL–BADL disability, and depressive symptoms (GDS ≥ 5) was 1.66 (95 % CI, 1.05–2.63, *p* = 0.031).

**Table 2:** Mortality (2004–2011) in SLAS cohort by living arrangement and other characteristics at baseline

	No (%) or mean (SD)	Person–years	Dead (*N* = 227)	Alive (*N* = 2384)	*P* value	Mortality rate per 1,000 p–y	Univariate HR (95 % C.I.)	
Total	2553	8062	227 (8.7)	2384 (91.3)		28.2		
Age (mean, SD)	66.04 (7.7)		73.27 (9.4)	65.35 (7.1)	<0.0001			
<75	2221	7014	131 (57.7)	2138 (89.7)	<0.0001	18.7	1.00	
75–84	282	884	68 (30.0)	221 (9.3)		76.9	3.88	(2.90–5.20)
85+	50	165	28. (12.3)	25. (1.0)		169.7	8.11	(5.37–12.25)
Female	1618	5095	99. (43.6)	1549. (65.0)	<0.0001	19.4	1.00	
Male	935	2967	128 (56.4)	835 (35.0)		43.1	2.36	(1.81–3.07)
Housing type: Higher end public and private	1780	5525	116 (51.1)	1706 (71.6)	<0.0001	21.0	1.00	
Low end (1–3 room) public	773	2537	111 (48.9)	678 (28.4)		43.8	1.51	(1.16–1.98)
No. medical comorbidities : ≤1	815	2631	46 (20.3)	787 (33.0)	<0.0001	17.5	1.00	
≥2	1738	5432	181 (79.7)	1597 (67.0)		33.3	2.06	(1.49–2.85)
History of Hypertension: No	1128	3549	74 (32.6)	1085 (45.5)	0.0002	20.9	1.00	
Yes	1425	4514	153 (67.4)	1299 (54.5)		33.9	1.54	(1.16–2.03)
History of Diabetes: No	2114	6653	164 (72.3)	1995 (83.7)	<0.0001	24.7	1.00	
Yes	439	1409	63 (27.8)	389 (16.3)		44.7	1.77	(1.33–2.37)
History of Chronic Lung Disease: No	2461	7781	211 (93.0)	2301 (96.5)	0.011	27.1	1.00	
Yes	92	268	16 (7.1)	78 (3.3)		59.7	2.19	(1.31–3.64)
History of Stroke: No	2459	7759	207 (91.2)	2303 (96.6)	<0.0001	26.7	1.00	
Yes	94	303	20 (8.8)	81 (3.4)		66.0	2.79	(1.76–4.43)
History of Heart Disease: No	2326	7313	180 (79.3)	2191 (91.9)	<0.0001	24.6	1.00	
Yes	227	730	47 (20.7)	186 (7.8)		64.4	2.50	(1.81–3.45)
History of Kidney Failure: No	2536	8009	225 (99.1)	2368 (99.3)	0.715	28.1	1.00	
Yes	17	53	2 (0.9)	16 (0.7)		37.7	1.25	(0.31–5.02)
IADL–BADL % (at least 1 item) disability: No	1926	6003	98 (43.2)	1872 (78.5)	<0.0001	16.3	1.00	
Yes	627	2059	129 (56.8)	512 (21.5)		62.6	3.06	(2.35–3.99)
Depressive symptoms (GDS > =5): No	2217	6892	178 (79.1)	2078 (87.4)	0.0005	25.8	1.00	
Yes	336	1141	47 (20.9)	300 (12.6)		41.2	1.39	(1.01–1.92)
Single, divorce, widowed: No	1909	6049	1802 (7.7)	141 (62.4)		23.3	1.00	
Yes	644	1997	578 (24.3)	85 (37.6)	<0.0001	42.6	1.93	(1.47–2.53)
Living alone: No	2364	7324	198 (89.6)	2166 (92.9)	0.044	27.0	1.00	
Yes	189	567	23 (10.4)	166 (7.1)		40.5	1.60	(1.04–2.46)

**Table 3:** Hierarchical Cox regression models of mortality associated with living alone: (mortality up to 31 Dec 2011)

		HR	95 % CI	*p*
Adjusted	Socio–demographic status (base model)	1.80	(1.16–2.78)	0.009
	Socio–demographic + health status	1.85	(1.17 –2.91)	0.008
	Socio–demographic + marital status	1.47	(0.93–2.32)	0.099
	Socio–demographic + health status + marital status	1.66	(1.05–2.63)	0.031

Socio–demographic status: age, sex and housing type

Health status: history of hypertension, diabetes, chronic lung disease, stroke, heart disease, kidney failure, IADL–BADL disability, GDS ≥ 5

Marital status: single, divorced/widowed versus married

In hierarchical models, the hazard ratio associated with living alone that controlled for sex, age and housing type in the base model (HR = 1.80) was not changed by the inclusion of health factors (HR = 1.84), but was substantially reduced by the inclusion of marital status (HR = 1.47).

In exploring possible effect modifications, no tests of statistical interactions were significant at *p* < 0.05. However, possible substantive effect modification by sex (*p* for interaction = 0.106) and marital group (*p* for interaction = 0.115) were observed. In stratified analyses, (Table 4), living alone was more strongly associated with mortality among men (HR = 2.36, 95 % CI, 1.24–4.49) than among women (HR = 1.14, 95 % CI, 0.58–2.22), *p* for interaction =0.106), and among single, divorced or widowed (HR = 2.26, 95 % CI, 1.24–4.10) than married individuals, (HR = 0.83, 95 % CI, 0.30–2.31), *p* for interaction = 0.115. Higher mortality associated with living alone were also observed for younger old (aged below 75 years), HR = 2.03, 95 % CI, 1.09–3.78), and those with no IADL–BADL disability (HR = 2.12, 95 % CI, 1.09–4.14), but with significant tests of statistical interaction at *p* > 0.15.

**Table 4:** Hazard ratios of association of living alone with mortality in stratified Cox regression analyses (mortality up to 31 Dec 2011)

		Adjusted for sex and age			Adjusted for all variables^a^			Interaction
	N	HR	95 % CI	p	HR	95 % CI	P	p
Male	935	2.53	(1.39–4.61)	0.0023	2.36	(1.24–4.49)	0.009	
Female	1618	1.15	(0.61–2.16)	0.664	1.14	(0.58–2.22)	0.705	0.106
Single, divorce, widowed	644	1.84	(1.06–3.18)	0.029	2.26	(1.24–4.10)	0.008	0.115
Married	1909	0.77	(0.28–2.11)	0.605	0.83	(0.30–2.31)	0.721	
Higher end housing	1780	1.50	(0.55–4.13)	0.430	1.32	(0.47–3.72)	0.600	
1–3 room public housing	773	1.61	(0.98–2.67)	0.063	1.67	(0.98–2.85)	0.058	0.600
<75 years	2221	2.69	(1.55–4.67)	0.0004	2.03	(1.09–3.78)	0.025	
≥75 years	332	0.78	(0.37–1.63)	0.509	1.09	(0.50–2.40)	0.825	0.377
No IADL–BADL disability	1926	2.57	(1.44–4.61)	0.0015	2.12	(1.09–4.14)	0.027	
IADL–BADL disability	627	1.00	(0.51–1.99)	0.993	1.21	(0.60–2.46)	0.595	0.305

IADL: Instrumental activity of daily living; BADL: Barthel Activity of daily living

^a^Age, sex, housing type, marital status, history of hypertension, diabetes, heart disease, chronic lung disease, stroke, kidney failure, IADL–ADL disability, and depressive symptoms (GDS ≥ 5), as appropriate

## Discussion

Here we found that living alone, independently of age, sex, socioeconomic, marital, and health status, was significantly associated with increased mortality overall among older persons living in Singapore. Older persons who live alone are recognized to be a vulnerable risk group in the population requiring special attention. Numerous studies show that elderly people living alone in the community are characterized by difficult living situations, limited resources and lack of support [45], and in need of medical services, financial subsidy and social and leisure activity setting [46]. The lack of informal and formal support of family members and social services in monitoring health condition, medical appointments [47] and caregiving [48, 49] is associated with poor self–management of chronic disease and increased risk of dying among the elderly who live alone.

Interestingly, in this population, we observed that older persons living alone, compared to their counterparts, did not have more medical morbidities or physical functional disability, which are established predictors of mortality. The increased mortality associated with living alone in this population could not therefore be attributed to poorer health and functional status, and adjustment for these health–related variables did not reduce the hazard ratio estimate. This finding is similar to that reported in the NHANES I Epidemiologic Follow Up Study in the United States which also found that older persons living alone did not differ from those living with others on the number of medical morbidities, and adjusting for the number of chronic conditions did not reduce the relative risk of mortality associated with living alone [20].

As expected, a substantially greater proportion of those living alone compared to those living with others were unmarried (single, widowed or divorced), which has been shown in this and many other studies to be associated with increased mortality [35]. The inclusion of marital status in the hierarchical model reduced the hazard ratio estimate of association of living alone with mortality. Being single, widowed or divorced was thus an important factor contributing to the increased mortality observed among older persons who love alone. However, our analysis controlling for confounding by marital status showed that living alone remained significantly and independently associated with increased mortality.

Prior studies have not uniformly shown that living alone was associated with increased mortality, as some studies have either reported no increased mortality [20–23] or paradoxically decreased mortality [24–26]. This suggests that the impact of living alone on mortality may be heterogeneous across different populations and studies [17, 18, 20, 23, 24, 28–31]. This is supported by data in this study. Although the significance tests were not significant for a hypothesized specific interaction at *p* < 0.05, substantively important interactions with *p* < 0.15 were possibly present, especially for sex and marital status, and should not be ignored.

Perhaps unsurprisingly, the increased mortality associated with living alone was clearly evident only among men, whereas among women, living alone was not significantly associated with increased mortality, consistent with findings in prior studies [5, 15–17]. Given the longer life expectancy of women, a survival cohort effect among women may explain this, but the difference between men and women in ability for self–care is another mechanistic explanation, and requires further study.

Mortality associated with living alone appeared to be particularly pronounced among those who were single, widowed or divorced, and not at all among those who were married. Thus, although living alone was shown to have a negative impact on mortality independently of the confounding influence of marital status, there was possible effect modification by marital status, with its mortality impact being exaggerated among those who were single, widowed or divorced. Living alone and being single, widowed or divorced may be viewed to represent complementing objective measures of social isolation and lack of social support, and together they thus appear to amplify the risk of dying among older persons. It is explicable that both factors share some common mediating biopsychosocial pathways in terms of the lack of both informal and formal support such as in maintaining adequate nutrition, medication adherence, monitoring health condition, keeping medical appointments, caregiving and social–emotional well–functioning which are related to health outcomes [50].

In agreement with prior studies [17, 18, 20], living alone was found in this study to be associated with higher mortality among younger participants, but among older persons (aged 75 years and over), living alone was not associated with increased mortality. This paradoxical finding may be explained by the surviving cohort effect in the oldest population group, who represent the remnants from prior mortality attrition at younger age of their peers. In the same vein, authors have pointed out that older persons who live alone are more likely to be a self–selected population of those who are in good health and independent in the basic activities of daily living. (28, 31)

Another paradoxical finding was that living alone was associated with increased mortality among those who were *without* physical functional disability; among those with physical functional disability, no increased mortality associated with living alone was found among those with physical functional disability. This is also likely to be explained by the self–selection process. As pointed out by previous authors, older persons with physical functional dependency are less likely to be found living alone and more likely to be found living with others [24]. Notably in Singapore, all elderly persons are identified in a watch list of vulnerable individuals for special attention and support by local voluntary befrienders and welfare workers. Because of this, it is possible that older people found to be in poor health or functionally dependent are likely to be placed with their family caregivers or in nursing homes.

In this study, we thus highlight dimensions of the relationship between living alone and mortality among older persons that remain unclear with findings from previous studies. We pointed out that in different study populations, variable selection characteristics of older persons living alone likely make for much heterogeneity of effect estimates of the relationship. Hence variable patterns of mortality risks associated with living alone may be expected across different study populations and internally among socio–demographic subgroups, especially by sex and marital status. Our results suggest that this is likely to be so, and should be further investigated in other population studies.

There are limitations in this study. Living arrangement is not an all–encompassing measure of social support. As a surrogate measure of many components of social support, it does not include a direct measure of resource deprivation for example, or the amount and quality of social contacts, and does not include subjective measures of perceived support or loneliness. As well, being unmarried was analysed as a surrogate for living without spousal support, but does not include a direct measure of the quality of spousal relationship. Further studies should elucidate the mechanisms and pathways through which social support influences health outcomes including mortality. Further studies should also investigate personal factors such as help–seeking behaviour, and its interactions with system factors of accessibility and effectiveness of health and social services in influencing the relationship between social isolation and health outcomes. For example, in some countries with well supported services effectively serving high risk vulnerable groups of elderly living alone, there may be no observable risk of excess mortality.

The study has strengths in examining a population–based cohort in an Asian setting that encompassed a heterogeneous mix of population characteristics, rather than a selected population of diseased or institutionalized individuals. With computerized record linkage to the National Death Registry, the ascertainment for the occurrence and date of death is virtually complete and accurate for all–cause mortality. The model estimates of the relationships between various risk factors and mortality were robust, from including multiple confounding co–variables in the models. Nevertheless, there remains a possibility of residual confounding by unmeasured variables such as cognitive status.

The findings in this study have important implications for rapidly ageing populations in Asia and elsewhere which are at various stages of socio–economic development while meeting the mounting challenges of healthcare and social services for their ageing population. For example, countries like China and Indonesia are “growing older without becoming rich”, whereas countries like Singapore. Taiwan and Korea are “growing older but are becoming rich”. Asian countries also differ substantially on their health and long–term care support systems for their elderly populations, among themselves and from Western countries. Given the scarcity of such studies in Asia, the excess mortality risk associated with living alone observed among elderly people living in Singapore is therefore noteworthy in the context of its high level of economic development, but facing challenges and dilemmas in providing long–term care to its escalating numbers of elderly people. The same challenges are faced by other Asian countries such as Taiwan or Korea which have national insurance for health services or long–term care.

## Conclusions

In conclusion, living alone was found to be associated with increased mortality, independently of marital, health and other variables. The impact of living alone on mortality appeared to be stronger among men and those who were single, divorced or married.

## References

[1] Tomassini C, Glaser K, Wolf DA, van Groenou MIB, Grundy E (2004). Living arrangement among older people: overview of trends in Europe and the USA. Population Trends.

[2] Martin LA (1990). Changing intergenerational family relations in East Asia. Ann Am Acad Pol Soc Sci.

[3] Frasure-Smith N, Lespérance F, Gravel G, Masson A, Juneau M, Talajic M, Bourassa MG (2000). Social support, depression, and mortality during the first year after myocardial infarction. Circulation.

[4] Kop WJ, Berman DS, Gransar H, Wong ND, Miranda-Peats R, White MD (2005). Social network and coronary artery calcification in asymptomatic individuals. Psychosom Med.

[5] Hirdes JP, Forbes WF (1992). The importance of social relationships, socioeconomic status and health practices with respect to mortality among healthy Ontario males. J Clin Epidemiol.

[6] Rees CA, Karter AJ, Young BA (2010). Race/ethnicity, social support, and associations with diabetes self–care and clinical outcomes in NHANES. Diabetes Educ.

[7] Rodriguez CJ, Elkind MS, Clemow L, Jin Z, Di Tullio M (2011). Association between social isolation and left ventricular mass. Am J Med.

[8] Christakis NA, Fowler JH (2007). The spread of obesity in a large social network over 32 years. N Engl J Med.

[9] Lund R, Due P, Modvig J, Holstein BE, Damsgaard MT, Andersen PK (2002). Cohabitation and marital status as predictors of mortality––an eight year follow–up study. Soc Sci Med.

[10] Koskinen S, Joutsenniemi K, Martelin T, Martikainen P (2007). Mortality differences according to living arrangements. Int J Epidemiol.

[11] Crockett AJ, Cranston JM, Moss JR, Alpers JH (2002). The impact of anxiety, depression and living alone in chronic obstructive pulmonary disease. Qual Life Res.

[12] Börü UT, Oztürk E, Taşdemir M, Sur H (2007). Living alone following first–ever stroke: a prospective study in Turkey identifying the risk factors and evaluating their effects. N Z Med J.

[13] Herttua K, Martikainen P, Vahtera J, Kivimäki M (2011). Living alone and alcohol–related mortality: a population–based cohort study from Finland. PLoS Med.

[14] Holwerda TJ, Beekman AT, Deeg DJ, Stek ML, van Tilburg TG, Visser PJ, et al. Increased risk of mortality associated with social isolation in older men: only when feeling lonely? Results from the Amsterdam Study of the Elderly (AMSTEL). Psychol Med. 2012;42(4):843–53. 10.1017/S0033291711001772 21896239

[15] Schmaltz HN, Southern D, Ghali WA, Jelinski SE, Parsons GA, King KM, et al. Living alone, patient sex and mortality after acute myocardial infarction. J Gen Intern Med. 2007;22(5):572–8. 10.1007/s11606-007-0106-7 PMC1852915 17443363

[16] Case RB, Moss AJ, Case N, McDermott M, Eberly S (1992). Living alone after myocardial infarction. Impact on prognosis. JAMA.

[17] Udell JA, Steg PG, Scirica BM, Smith SC, Ohman EM, Eagle KA, Goto S, Cho JI, Bhatt DL, REduction of Atherothrombosis for Continued Health (REACH) Registry Investigators (2012). Living alone and cardiovascular risk in outpatients at risk of or with atherothrombosis. Arch Intern Med.

[18] Gopinath B, Rochtchina E, Anstey KJ, Mitchell P. Living alone and risk of mortality in older, community–dwelling adults. JAMA Intern Med. 2013;173(4):320–1. 10.1001/jamainternmed.2013.1597 23319114

[19] Perissinotto CM, Stijacic Cenzer I, Covinsky KE (2012). Loneliness in older persons: a predictor of functional decline and death. Arch Intern Med.

[20] Davis MA, Neuhaus JM, Moritz DJ, Segal MR (1992). Living arrangements and survival among middle–aged and older adults in the NHANES I epidemiologic follow–up study. Am J Public Health.

[21] Steinbach U (1992). Social networks, institutionalization, and mortality among elderly people in the United States. J GerontoL.

[22] Wolinsky FD, Johnson RL, Stump TE (1995). The risk of mortality among older adults over an eight–year period. Gerontologist..

[23] Simons LA, McCallum J, Simons J (2013). Impact of loneliness and living alone. JAMA Intern Med.

[24] Jagger C, Clarke M (1988). Mortality risks in the elderly: five–year follow–up of a total population. Int J Epidemiol.

[25] Murata C, Takaaki K, Hori Y, Miyao D, Tamakoshi K, Yatsuya H, et al. Effects of social relationships on mortality among the elderly in a Japanese rural area: an 88–month follow–up study. J Epidemiol. 2005;15(3):78–84. 10.2188/jea.15.78 PMC7851063 15930803

[26] Rozzini R, Trabucchi M (2013). Health status in elderly persons living alone. JAMA Intern Med.

[27] Holt–Lunstad J, Smith TB, Baker M, Harris T, Stephenson D (2015). Loneliness and social isolation as risk factors for mortality: a meta–analytic review. Perspect Psychol Sci.

[28] Hanson BS, Isacsson SO, Janzon L, Lindell SE (1989). Social network and social support influence mortality in elderly men. Am J Epidemio.

[29] Kandler U, Meisinger C, Baumert J, Lowel H (2007). Living alone is a risk factor for mortality in men but not women from the general population: a prospective cohort study. BMC Public Health.

[30] Staehelin K, Schindler C, Spoerri A, Zemp Stutz E. Marital status, living arrangement and mortality: does the association vary by gender? J Epidemiol Community Health. 2011;66(7):e22. 10.1136/jech.2010.128397 22012962

[31] Covinsky KE (2013). The differential diagnosis of living alone: comment on “Living alone and risk of mortality in older, community–dwelling adults”. JAMA Intern Med.

[32] Umberson D (1992). Gender, marital status and the social control of health behavior. Soc Sci Med.

[33] Miller–Tutzauer C, Leonard KE, Windle M (1991). Marriage and alcohol use: A longitudinal studyof maturing out. J Stud Alcohol.

[34] Grundy EM, Tomassini C (2010). Marital history, health and mortality among older men and women in England and Wales. BMC Public Health.

[35] Manzoli L, Villari P, M Pirone G, Boccia A (2007). Marital status and mortality in the elderly: a systematic review and meta–analysis. Soc Sci Med.

[36] Niti M, Yap KB, Kua EH, Tan CH, Ng TP (2008). Physical, social and productive leisure activities, cognitive decline and interaction with APOE–epsilon4 genotype in Chinese older adults. Int Psychogeriatr.

[37] Department of Statistics, Ministry of Trade and Industry, Republic of Singapore. Census of Population 2010 Statistical Release 2: Households and Housing. http://www.singstat.gov.sg/publications/publications–and–papers/cop2010/census10_stat_release2#sthash.rEF1KOMS.dpuf

[38] National Cholesterol Education Program (NCEP) Expert Panel on Detection, Evaluation, and Treatment of High Blood Cholesterol in Adults (Adult Treatment Panel III). Third report of the National Cholesterol Education Program (NCEP) expert panel on detection, evaluation, and treatment of high blood cholesterol in adults (Adult Treatment Panel III). Final report. Circulation. 2002;106:3143–3421. 12485966

[39] Sheikh JI, Yesavage JA, Brink TL (1986). Geriatric Depression Scale (GDS): recent evidence and development of a shorter version. Clinical gerontology: a guide to assessment and intervention.

[40] Nyunt MSZ, Jin AZ, Fones CSL, Ng TP (2009). Criterion–based validity and reliability of the Geriatric Depression Screening Scale (GDS–15) in a large validation sample of community–living Asian older adults. Aging Ment Health.

[41] Collin C, Wade DT, Davies S, Horne V (1988). The Barthel ADL Index: a reliability study. Int Disabil Stud.

[42] Mp L, Brody EM (1969). Assessment of older people: self–maintaining and instrumental activities of daily living. Gerontologist.

[43] Ng TP, Niti M, Chiam PC, Kua EH (2006). Physical and cognitive domains of the instrumental activities of daily living: validation in a multiethnic population of Asian older adults. J Gerontol Biol Med Sci.

[44] Niti M, Ng TP, Chiam PC, Kua EH (2007). Item response bias was present in Instrumental Activity of Daily Living Scale in Asian older adults. J Clin Epidemiol.

[45] Haslbeck JW, McCorkle R, Schaffer D (2012). Chronic illness Self–Management While Living Alone in Later Life: A Systematic Integrative Review. Res Aging.

[46] Huang LH, Lin YC (2002). The health status and needs of community elderly living alone. J Nurs Res.

[47] Prohaska TR, Glasser M (1996). Patients’ views of family involvement in medical care decisions and encounters. Res Aging.

[48] Stone R, Cafferata GL, Sangl J (1987). Caregivers of the frail elderly: A national profile. Gerontologist.

[49] Burnette D, Riessman CK (1993). Managing chronic illness alone in late life: Sisyphus at work. Qualitative studies in social work research.

[50] Bucholz EM, Krumholz HM (2012). Loneliness and Living Alone: What Are We Really Measuring?. Arch Intern Med.

